# Renal artery aneurysm presenting with severe hematuria: a case report

**DOI:** 10.11604/pamj.2026.53.22.50469

**Published:** 2026-01-16

**Authors:** Ousmane Sow, Cyrille Ze Ondo, Momar Sokhna Diop

**Affiliations:** 1 Urology-Andrology Department, Aristide Le Dantec Hospital, Dakar, Senegal,; 2 Department of Thoracic and Cardiovascular Surgery, Fann University Hospital, Dakar, Senegal

**Keywords:** Renal artery, aneurysm, hematuria, embolization, case report

## Abstract

Renal artery aneurysm is rare, and its incidence in the general population remains elusive. Most aneurysms are asymptomatic. When symptomatic, renal artery aneurysm may be associated with hypertension, flank pain, hematuria, or urinary collecting system obstruction. We report the case of a left renal artery aneurysm in a young adult. The symptomatology was marked by massive hematuria associated with left flank pain. A left subcapsular nephrectomy after a failed embolization attempt was performed. The postoperative course was uneventful.

## Introduction

Renal artery aneurysm (RAA) is defined as a dilated segment of renal artery that exceeds twice the diameter of a normal renal artery [1]. Its incidence ranges from 0.3% to 1% in patients undergoing imaging studies for unrelated conditions [2]. Most aneurysms are asymptomatic. When symptomatic, RAA may be associated with hypertension, flank pain, hematuria, or urinary collecting system obstruction. The treatment of symptomatic RAA involves endovascular techniques or open surgical repair, depending on aneurysm size, morphology and location. We report the case of a left renal artery aneurysm with massive hematuria in a young adult.

## Patient and observation

**Patient information:** a 35-year-old man, with no past medical and surgical history, presented to the urological emergency room with massive hematuria and sharp left flank pain radiating posteriorly. He had no nausea, vomiting, or fever. He had experienced three episodes of left flank pain over the last 6 months.

**Clinical findings:** physical examinations revealed tenderness in the left epigastric region and percussion pain in the left kidney region. The blood pressure was normal.

**Figure 1:**
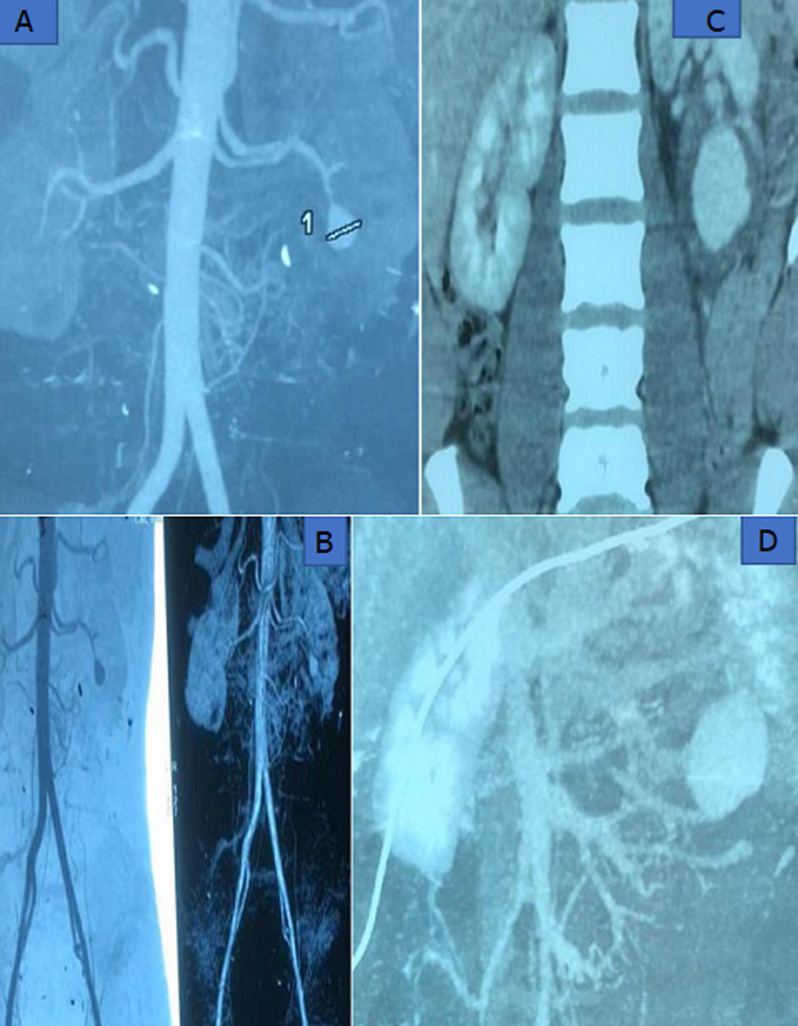
A, B) abdominal computed tomography angiography revealed an aneurysm of the left inferior polar segmental artery measuring 42 in height and 32x33 mm in transverse axes; (C, D) the aneurysm was surrounded by a circumferential hematoma measuring 12.7 mm in thickness

**Diagnostic assessment:** laboratory tests evaluating renal function and blood film results were normal. During one of the episodes, the patient had undergone an abdominal ultrasound, which showed a left renal intraparenchymal aneurysm about 16 mm in diameter. Abdominal computed tomography angiography revealed an aneurysm of the left inferior polar segmental artery measuring 42 in height and 32x33 mm in transverse axes. The aneurysm was surrounded by a circumferential hematoma measuring 12.7 mm in thickness (Figure 1).

**Figure 2:**
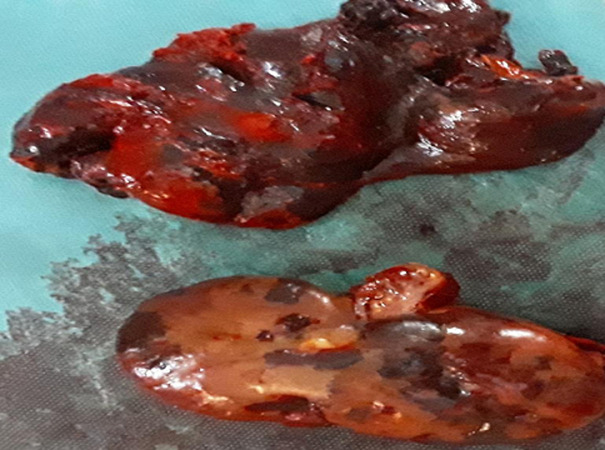
post-operative picture of the subcapsular nephrectomy

**Therapeutic interventions:** selective arterial embolization was decided by a multidisciplinary consultation that included vascular surgeons, urologists, and interventional radiologists. Four days after selective arterial embolization, clinical symptoms did not improve with persistent massive hematuria and refractory hypotension. Blood pressure was 90/60 mmHg and hemoglobin was 7.5 g/dl. Thus, in front of this clinical presentation, we decided to perform a total nephrectomy. Blood transfusions were done preoperatively. A subcapsular nephrectomy was performed (Figure 2).

**Follow-up and outcome of interventions:** postoperative course was uneventful and the patient was discharged on the 5^th^ postoperative day in good physical condition with hemoglobin 11.6 g/dl and creatinine 10.8 mg/L. Exactly, 7 months after operation, the patient is well with normal blood pressure and renal function.

**Patient perspective:** the patient was delighted with the quality of care.

**Informed consent:** written informed consent was obtained from the patient for participation in our study.

## Discussion

RAAs are uncommon and predominantly asymptomatic. Angiographic and computed tomography studies report an incidence from 0.3% to 2.5% [2]. RAAs typically present in the sixth decade, unlike our young patient. RAAs have been classified, according to their shape, as saccular, fusiform, dissecting, and microaneurysms [3]. Women are more commonly afflicted with renal artery aneurysm, likely due to the high incidence of associated fibromuscular dysplasia [3].

Clinical manifestations of RAAs vary from being asymptomatic to fatal rupture. RAAs may be detected incidentally as well as present with urologic symptoms and signs related to complications [3]. Hematuria may be due to a perforation of the RAA into the collecting system or to embolic renal infarct. As reported in our case, hematuria may occur for aneurysm impressing and congesting both the calyceal system and pelvis of the kidney. Flank or abdominal pain and massive hematuria may be secondary to renal artery aneurysm rupture with retroperitoneal hemorrhage, as well as renal infarction. RAA rupture may be retroperitoneal and intrarenal. The finding of abdominal mass or costovertebral tenderness associated with pain, hematuria and acute hypotension may indicate RAA rupture with a large retroperitoneal hemorrhage formation. RAAs can present with massive hematuria and are potentially life-threatening in cases of rupture.

RAAs are investigated by non-invasive modalities including duplex ultrasound, magnetic resonance angiography, and spiral three-dimensional computed tomography angiography [4]. All these imaging modalities can provide helpful information for diagnosing and planning treatment of RAA. Angiography with intra-arterial injection of contrast material is considered the gold standard before performing any treatment since it confirms the presence as well as provides anatomical localization and assessment of the aneurysms or pseudoaneurysms.

The treatments for RAA include endovascular repair, such as selective coil embolization or stenting, and surgical repair. Nephrectomy was necessary in cases of complex intraparenchymal aneurysms, multiple aneurysms, or ruptured aneurysms. Endovascular interventions include stent-graft exclusion of the aneurysm, simple coil embolization of the aneurysm, stent-coiling, and coil occlusion with the sacrifice of the aneurysm parent artery. The need for a nephrectomy has virtually disappeared in the current era. However, in the setting of early failures of repair, persistent bleeding, refractory hypotension, unreconstructable renal arteries, and if there is normal contralateral kidney, nephrectomy was indicated [5]. Our patient had persistent bleeding with refractory hypotension after early failure of elective arterial embolization. We opted for a total nephrectomy and the postoperative course was uneventful.

## Conclusion

RAA is a rare disorder. It is difficult to reach a consensus on the appropriate indications for intervention. RAA can bleed into his collecting system or rupture and cause a life-threatening hemorrhage, hence the need for early and appropriate management.
